# Non‐pharmacological interventions for older adults with early cognitive impairment

**DOI:** 10.1002/alz70858_096791

**Published:** 2025-12-24

**Authors:** Natanya S Russek, Philip E Lee

**Affiliations:** ^1^ University of British Columbia, Vancouver, BC, Canada

## Abstract

**Background:**

As the population ages, mild cognitive impairment (MCI) and dementia are increasingly prevalent. Patients, families, and healthcare systems will benefit from delaying progression of cognitive decline. Non‐pharmacological interventions (NPIs) are safe, well‐tolerated, and preferred by patients and have been studied in a broad body of literature. Despite this, few comprehensive guidelines exist on NPIs for early cognitive decline.

**Methods:**

We conducted a systematic review of the literature focused on NPI supported by the FINGER model and a review conducted by the AAIC Non‐Pharmacological Interventions Professional Interest Area (Sikkes et al 2021). Inclusion criteria included meta‐analysis or systematic review of randomized controlled trials enrolling patients ≥ 60 with MCI or dementia (MMSE >20 when provided). Included studies were published between 2014 ‐ 2024, and outcomes assessed included cognition and/or function.

**Results:**

Of 2,870 studies screened, 26 met inclusion criteria: exercise (*n* = 16), cognitive (*n* = 4), multicomponent (*n* = 5), and mindfulness (*n* = 1) interventions. Exercise, including dance, exergames, and mind‐body showed improvement in global and cognitive sub‐domains but benefit was not seen with walking alone. Cognitive interventions, including cognitive stimulation, mindfulness, and multicomponent interventions showed cognitive benefits. Only one study of virtual reality cognitive training improved functional status. Considerable heterogeneity including variable trial duration, multiple interventions studied, and inability to fully blind participants and researchers limit comparability and conclusions.

**Conclusions:**

NPIs benefit cognition in individuals with MCI and mild dementia. The most benefit was demonstrated with multi‐domain interventions incorporating cognitive and exercise interventions, mindfulness, and some cognitive interventions. These interventions can be accessed through community or senior centres or integrated into clinic settings with multidisciplinary care teams. We recommend an individualized approach for each patient, which incorporates their interests, frailty, mobility, medical comorbidity, and level of function. Further research is needed to inform specific recommendations about how much cognitive or physical activity is optimal for improving cognition or function.

